# Supplementary material for: Predictive design of sigma factor-specific promoters
Source: Nat Commun. 2020 Nov 16;11:5822. doi: 10.1038/s41467-020-19446-w (PMC7670410; doi:10.1038/s41467-020-19446-w)
Supplement: Supplementary file 1 — Supplementary Information [file 41467_2020_19446_MOESM1_ESM.pdf]

SUPPLEMENTARY DATA OF PREDICTIVE DESIGN OF SIGMA FACTOR-SPECIFIC PROMOTERS

Van Brempt Maarten[1 †], Jim Clauwaert[2 †], Friederike Mey[1], Michiel Stock[2], Jo Maertens[1], Willem Waegeman[2 ‡] and Marjan De Mey[1 * ‡].

[1] Centre for Synthetic Biology (CSB), Department of Biotechnology, Ghent University, 9000 Ghent, Belgium.

[2] KERMIT, Department of Data Analysis and Mathematical Modelling, Ghent University, 9000 Ghent, Belgium

* To whom correspondence should be addressed. Tel: +32 9 264 6028
Email: marjan.demey@ugent.be

[†] These authors contributed equally to the paper as first authors.

[‡] These authors contributed equally to the paper as last authors.

Supplementary Table 1: Counts of the reads and unique sequences in each bin and amount of sequences assigned to each class, before and after subsetting the data. For each library the genotypic background is given: heterologous sigma factors (σ) B, F or W from *Bacillus subtilis* in *Escherichia coli* (*E. coli*), or wild-type (WT) *E. coli* harbouring no heterologous σs. Source data are provided as a Source Data file.

		**Reads**			**Unique sequences**			**Labels**		
	**Class**	**Original**	**Filtered**	**Fraction**	**Original**	**Filtered**	**Fraction**	**Original**	**Filtered**	**Fraction**
**<u>σ^{70} specific promoter library</u>**										
**WT genotype**										
**Bin 0**	-	8,869	3,079	0.347	4,997	1,926	0.385	2,144	1,341	0.625
**Bin 1**	0	12,489	3,735	0.299	4,193	1,561	0.372	1,832	1,091	0.596
**Bin 2**	1	26,591	10,052	0.378	12,091	5,310	0.439	4,760	2,988	0.628
**Bin 3**	2	64,493	29,591	0.459	22,423	12,657	0.564	10,624	7,716	0.726
**Bin 4**	3	213,448	111,248	0.521	50,361	34,536	0.686	34,649	25,638	0.740
**Bin 5**	4	259,482	184,601	0.711	102,643	83,310	0.812	59,649	53,975	0.905
**Bin 6**	5	287,517	223,511	0.777	115,554	99,036	0.857	72,723	68,156	0.937
**Bin 7**	6	249,955	195,856	0.784	100,934	86,744	0.859	66,246	62,028	0.936
**Bin 8**	7	139,423	108,440	0.778	49,862	42,161	0.846	36,539	33,755	0.924
**Bin 9**	8	66,854	51,312	0.768	32,895	26,842	0.816	23,273	21,708	0.933
**Bin 10**	9	15,068	10,681	0.709	8,016	6,076	0.758	5,742	5,107	0.889
**Bin 11**	10	42,425	9,836	0.232	3,468	2,283	0.658	3,394	2,259	0.666
**Total**		1,386,614	941,942	0.679	507,437	402,442	0.793	321,575	285,762	0.889
**<u>σ^{B} specific promoter library</u>**										
**σ^{B} genotype**										
**Bin 0**	-	74,764	48,727	0.652	39,261	28,015	0.714	22,360	19,071	0.853
**Bin 1**	0	48,848	35,475	0.726	25,256	19,651	0.778	13,474	12,091	0.897
**Bin 2**	1	135,710	95,624	0.705	57,957	45,753	0.789	38,290	33,252	0.868
**Bin 3**	2	37,287	29,372	0.788	27,939	22,696	0.812	11,547	11,151	0.966
**Bin 4**	3	146,443	111,680	0.763	70,082	57,933	0.827	45,562	41,625	0.914
**Bin 5**	4	83,237	64,452	0.774	43,666	35,508	0.813	26,566	24,350	0.917
**Bin 6**	5	21,618	16,344	0.756	14,647	11,315	0.773	7,356	6,799	0.924
**Bin 7**	6	26,203	19,088	0.728	13,365	10,108	0.756	8,071	7,217	0.894
**Bin 8**	7	22,843	15,623	0.684	9,628	6,934	0.720	6,274	5,284	0.842
**Bin 9**	8	20,725	11,827	0.571	6,007	4,083	0.680	4,345	3,372	0.776
**Bin 10**	9	27,383	12,536	0.458	6,279	3,819	0.608	4,995	3,450	0.691
**Bin 11**	10	25,958	1,078	0.042	1,504	691	0.459	1,383	658	0.476
**Total**		671,019	461,826	0.688	315,591	246,506	0.781	190,223	168,320	0.885
**σ^{F} genotype**										
**Bin 0**	0	362,520	301,168	0.831	150,625	140,930	0.936	139,867	131,102	0.937
**Bin 1**	1	83,078	68,320	0.822	46,188	40,458	0.876	32,807	30,515	0.930
**Total**		445,598	369,488	0.829	196,813	181,388	0.922	172,674	161,617	0.936
**σ^{W} genotype**										
**Bin 0**	0	481,962	383,810	0.796	168,386	156,131	0.927	159,443	147,771	0.927
**Bin 1**	1	76,485	62,619	0.819	41,656	36,430	0.875	26,409	25,394	0.962
**Total**		558,447	446,429	0.799	210,042	192,561	0.917	185,852	173,165	0.932
**WT genotype**										
**Bin 0**	0	325,625	274,145	0.842	142,415	133,503	0.937	126,596	119,258	0.942
**Bin 1**	1	116,015	95,893	0.827	61,645	55,644	0.903	44,845	42,648	0.951
**Total**		441,640	370,038	0.838	204,060	189,147	0.927	171,441	161,906	0.944

**σ^F specific promoter library**										
**σ^F genotype**										
**Bin 0**	-	90,832	61,343	0.675	53,157	38,868	0.731	32,411	26,829	0.828
**Bin 1**	0	65,860	47,317	0.718	38,090	29,495	0.774	22,912	19,696	0.860
**Bin 2**	1	97,645	73,643	0.754	61,162	49,374	0.807	35,627	31,450	0.883
**Bin 3**	2	131,183	103,970	0.793	82,406	69,634	0.845	49,523	45,353	0.916
**Bin 4**	3	129,678	105,467	0.813	86,032	73,331	0.852	52,522	48,112	0.916
**Bin 5**	4	172,479	133,382	0.773	91,638	75,932	0.829	66,326	59,240	0.893
**Bin 6**	5	21,047	15,086	0.717	17,955	12,899	0.718	9,940	7,674	0.772
**Bin 7**	6	19,240	13,856	0.720	12,552	9,315	0.742	7,449	6,460	0.867
**Bin 8**	7	12,617	7,804	0.619	7,964	4,963	0.623	5,294	3,767	0.712
**Bin 9**	8	9,251	1,938	0.209	1,778	735	0.413	1,602	695	0.434
**Bin 10**	9	25,692	1,496	0.058	2,308	1,425	0.617	1,955	1,384	0.708
**Bin 11**	10	34,529	693	0.020	643	494	0.768	557	486	0.873
**Total**		810,053	565,995	0.699	455,685	366,465	0.804	286,118	251,146	0.878
**σ^B genotype**										
**Bin 0**	0	175,309	153,129	0.873	82,651	78,100	0.945	74,016	70,096	0.947
**Bin 1**	1	43,088	36,498	0.847	26,422	23,916	0.905	18,514	17,591	0.950
**Total**		218,397	189,627	0.868	109,073	102,016	0.935	92,530	87,687	0.948
**σ^W genotype**										
**Bin 0**	0	605,774	477,135	0.788	257,146	229,908	0.894	249,453	229,752	0.921
**Bin 1**	1	50,997	27,082	0.531	35,435	19,589	0.553	25,890	17,822	0.688
**Total**		656,771	504,217	0.768	292,581	249,497	0.853	275,343	247,574	0.899
**WT genotype**										
**Bin 0**	0	743,986	597,648	0.803	263,897	237,202	0.899	256,747	237,041	0.923
**Bin 1**	1	49,485	23,428	0.473	33,594	17,168	0.511	21,032	13,671	0.650
**Total**		793,471	621,076	0.783	297,491	254,370	0.855	277,779	250,712	0.903
**σ^W specific promoter library**										
**σ^W genotype**										
**Bin 0**	-	8,742	3,511	0.402	6,615	2,832	0.428	2,797	2,280	0.815
**Bin 1**	0	1,281	626	0.489	1,044	529	0.507	483	395	0.818
**Bin 2**	1	1,447	490	0.339	1,174	391	0.333	596	238	0.399
**Bin 3**	2	1,450	694	0.479	927	513	0.553	370	293	0.792
**Bin 4**	3	14,937	9,704	0.650	10,144	7,183	0.708	4,239	3,436	0.811
**Bin 5**	4	197,405	139,799	0.708	77,150	64,070	0.830	48,871	44,470	0.910
**Bin 6**	5	266,914	186,146	0.697	95,728	80,358	0.839	65,572	58,022	0.885
**Bin 7**	6	119,043	81,773	0.687	51,722	40,618	0.785	27,732	25,062	0.904
**Bin 8**	7	95,857	67,373	0.703	50,804	39,785	0.783	25,027	22,933	0.916
**Bin 9**	8	105,467	70,864	0.672	43,369	33,212	0.766	24,393	21,445	0.879
**Bin 10**	9	25,877	16,784	0.649	16,912	11,424	0.675	7,412	6,331	0.854
**Bin 11**	10	15,507	7,540	0.486	5,072	2,747	0.542	3,023	2,186	0.723
**Total**		853,927	585,304	0.685	360,661	283,662	0.787	210,515	187,091	0.889
**σ^B genotype**										
**Bin 0**	0	718,105	530,355	0.739	103,970	95,441	0.918	102,148	93,648	0.917
**Bin 1**	1	41,156	31,292	0.760	24,372	19,674	0.807	4,884	4,320	0.885
**Total**		759,261	561,647	0.740	128,342	115,115	0.897	107,032	97,968	0.915
**σ^F genotype**										
**Bin 0**	0	1,435,610	955,999	0.666	190,344	168,039	0.883	187,397	167,954	0.896
**Bin 1**	1	53,241	23,097	0.434	31,305	16,783	0.536	7,327	4,393	0.600
**Total**		1,488,851	979,096	0.658	221,649	184,822	0.834	194,724	172,347	0.885
**WT genotype**										
**Bin 0**	0	539,145	407,605	0.756	149,488	139,119	0.931	145,526	135,224	0.929
**Bin 1**	1	34,047	26,745	0.786	22,809	18,928	0.830	9,384	9,223	0.983
**Total**		573,192	434,350	0.758	172,297	158,047	0.917	154,910	144,447	0.932

Supplementary Table 2: Overview of all forward engineered promoter spacers, together with the predicted promoter transcription initiation frequency (TIF) class (0 – 10 and 'high'), its hamming distance to the closest sequence in the data set and the measured promoter TIF in terms of corrected fluorescence (see also Supplementary Figure 7). Source data are provided as a Source Data file.

ID	Spacer sequence (5' → 3')	Class	Hamming distance	Fluorescence, corrected
1	CTGATTTTAAGGGTTTA	0	2	0.032
2	GTATTCTTTCGTGTTTT	0	3	0.025
3	TTAAATTCACATCTTTT	0	3	0.039
4	GCATATTAGGCGATCAA	0	4	0.043
5	CTAAACCTTCCAATTTT	0	4	0.022
6	CTTAACTTCCGCATTTT	0	5	0.017
9	ATAGTGCAGGCCCTTTT	0	3	0.047
7	CAAATTTCCTCGGTTCC	1	2	0.029
10	CCCCACAATCACCTAAT	1	4	0.078
11	CGCTATTTAGCAGACTT	1	4	0.063
12	CCGCTGGCCACCAATTA	1	5	0.036
8	TTATTGTTGCAGGAATT	2	3	0.095
15	CAACTAATCTATATTTG	2	3	0.267
17	TAATTCAACTCCCTCCA	2	4	0.108
18	GCCTCTCTACCCACCCT	3	5	0.089
13	CCTGGAATGGAGCATCG	4	2	0.136
14	TGGTTAAGTGGTATAAG	4	3	0.399
16	GGGTTTGAAGGAAAAGA	4	4	0.427
24	CCTAAATGACCCTAGGT	5	5	0.234
20	GTTGTGCGTTGATTTCC	6	3	0.251
23	AGGTCTAAATCAGTTAT	6	4	0.344
29	CAGGTGGACCGTTAAGA	6	4	0.640
32	GAAGAGGTTGACCAGTC	6	3	0.208
21	CTTCCAACGCTTCGGCA	7	3	0.465
22	GTTGCCTACGATGAGAG	7	4	0.645
31	CGGACATACATTTAGCC	7	2	0.913
34	AGGAGTCCAACTTTCTC	7	4	1.281
37	GACTATCAAAAGTTTCC	7	3	1.033
19	GAGCCCCCTAGGACGGG	8	2	1.038
26	TTTGTGCAGTGGGAGGT	8	3	0.794
28	CCTAAAACCATCTAGGC	8	4	0.411
30	ATTAGCATCCCAGTAGT	8	5	0.780
35	CATTTTAATATCTAGCC	8	4	0.413
25	TTGGGTATTTGTGTATC	9	2	0.278
27	ATTGAGCCCAACTTTCA	9	3	0.546
33	TACAGTATGGTCGTGGC	9	3	0.462
36	CCTAGAAAACACAGATG	9	5	0.728

38	ATCCGTTAATGACCAGT	9	3	1.141
42	CACCGTCAGTCATCTAC	9	5	0.980
46	CATGGTTTTTATCGTTC	9	4	0.822
50	TTAGCCGATTCTTTAGC	9	5	1.881
39	TCGAAGTTAAACTCATA	10	3	1.466
40	CACACGCTAATTACTCC	10	4	0.665
41	ACTAGTGATACGTTTGT	10	4	2.127
43	GCACGTCACGTATGGGT	10	3	1.485
44	TATCTAAAAAGTTTTGG	10	3	1.017
45	CATTGTCTTTCATGCTA	10	3	1.181
49	CGGGCACATTTCCTTGG	10	3	1.285
47	TCGTGATAATTTGTGCA	high	4	1.895
48	TACGGCCATCCTTGTTG	high	5	1.476
51	TTAGCGAATTGTTTTGG	high	4	1.504
52	TCCACATTTTCTTTTGC	high	4	1.744
53	GGTCGGAAATTGCTTGC	high	4	2.022
54	AAAGAGGATTTGCTTGC	high	4	1.278

Supplementary Table 3: Model predictions for class of promoter transcription initiation frequency and orthogonality, for the defined sets of previously created promoters. Promoters containing deviant spacer lengths were excluded. Promoters are ordered ascending per sigma factor specificity, according to the measured *in vivo* expression level. Higher orthogonality parameter values indicate a higher probability for loss of orthogonality. Predictions with '*' indicate the specific sequence was part of the training data set. (NA: not applicable; WT: wild-type). Source data are provided as a Source Data file.

**Promoter ID**	**Spacer sequence (5' → 3')**	**Predicted Class**	**Orthogonality parameter versus genotypes:**			
			**σ^B**	**σ^F**	**σ^W**	**WT**
B1	CAAATGGTGCTG	0		0,36	0,50	0,48
B2	CGTTTAATCTGT	1 *		0,95	0,93	0,96 *
B3	AGGTCCTCAATT	4		0,25	0,15	0,20
B4	ATGTGCCTTTTG	9 *		0,29	0,29	0,28
B5	TCCCCAGTTTTG	9	NA	0,22	0,22	0,38
B6	TTGTTCGAAAGG	10 *		0,31	0,34	0,44
B7	CATATGCAAAAC	10		0,70	0,30	0,52
B8	TCTGGGAAAATC	10		0,61	0,49	0,86
B9	CTGTGGTAAAAC	10		0,95	0,96 *	0,72
F1	AGCTATTGAGGGTATT	2 *	0,32		0,34	0,26
F2	TGCCAAATGGCAGGTG	0	0,34		0,29	0,22
F3	TTGACGGATATCGCTG	0	0,24		0,28	0,11
F4	GTGATGTGTCACGATG	1 *	0,24		0,36	0,24
F5	TTTGAAGGGATGAGTG	4	0,41	NA	0,36	0,18
F6	GTTTTAATTATAACTG	8	0,62		0,59	0,72
F7	AAAACGATGCGTTGTG	9	0,71		0,49	0,26
F8	CATAATTTAATTTTGG	9 *	0,97		0,98	0,96
F9	CTTTTATGTGTTTATG	9	0,97		0,94	0,96
W4	CTTTTGAAAGGATTTG	0	0,41	0,19		0,38
W5	CTTTTGAACGTTTGCA	2 *	0,38	0,28		0,36
W6	GGAAAAATGGAGCGGG	9	0,39	0,27	NA	0,52
W7	CGATCGTCTGCGGACG	7	0,40	0,31		0,41
W8	GCGGAAAAACGAAGCT	10 *	0,22	0,08		0,21
W9	GTCTCGGAGGGGTGTT	7	0,31	0,40		0,38

Supplementary Table 4: Custom primers used in the first PCR step for Illumina sequencing sample preparation. Sequences in bold are the primer binding sites, specific for each type of promoter (sigma factor 70 (σ^{70}), σ^{B}, σ^{F} and σ^{W}-specific). 5' overhang includes linker sequences; which serve as template for the barcoding primers. Source data are provided as a Source Data file.

Primer name	Sequence (5' → 3')
oNGS_shared_ i7-adapter	GTCTCGTGGGCTCGGAGATGTGTATAAGAGACAG**GGCTCACAGAGTATAAGTGG**
oNGS_sigB_ i5-adapter	TCGTCGGCAGCGTCAGATGTGTATAAGAGACAG**GCCCTACAAGTTACATACCC**
oNGS_sigF_ i5-adapter	TCGTCGGCAGCGTCAGATGTGTATAAGAGACAG**CCTGTTTCTACTATTATGAG**
oNGS_sigW_ i5-adapter	TCGTCGGCAGCGTCAGATGTGTATAAGAGACAG**GCCCTCTGTATGTATACG**
oNGS_sig70_ i7-adapter	GTCTCGTGGGCTCGGAGATGTGTATAAGAGACAG**TTTGATCAGCTCGCTAACC**
oNGS_sig70_ i5-adapter	TCGTCGGCAGCGTCAGATGTGTATAAGAGACAG**TTGCTGGATAACTTTACG**

Supplementary Table 5. Performances of four of the evaluated model architectures for each of the four different data sets (sigma factor 70 (σ^{70}), σ^{B}, σ^{F} and σ^{W}-specific promoters) in order to select the optimal model architecture. The architectures were selected as they represent varying levels of complexity. The four architectures are a single node with no activation function, equaling linear regression (A1), a fully connected layer (A2), two fully connected layers of 128 and 64 nodes (A3), and the convolutional neural network used in the paper (A4). Every architecture has the ordinal layer as explained in the work. The best model architecture per metric is given in bold. More complex models did not yield better results. (WL: weighted loss; WMAE: weighted mean absolute error). Source data are provided as a Source Data file.

Data set	Model architecture	WL	WMAE	Accuracy	Spearman's rho
**σ^{70}**	A1	0.082	1.829	0.190	0.508
	A2	0.082	1.827	0.193	0.509
	A3	0.078	1.659	0.216	0.563
	A4	**0.076**	**1.626**	**0.230**	**0.572**
**σ^{B}**	A1	0.122	1.838	0.196	0.533
	A2	0.121	1.845	0.196	0.534
	A3	0.114	1.662	0.230	0.541
	A4	**0.112**	**1.638**	**0.232**	**0.568**
**σ^{F}**	A1	0.051	2.128	0.147	0.457
	A2	0.052	2.133	0.149	0.456
	A3	**0.048**	1.921	0.226	0.490
	A4	**0.048**	**1.903**	**0.228**	**0.505**
**σ^{W}**	A1	0.031	2.507	0.128	0.249
	A2	**0.030**	2.482	0.126	**0.260**
	A3	0.031	**2.438**	**0.133**	0.254
	A4	0.031	2.536	0.121	0.215

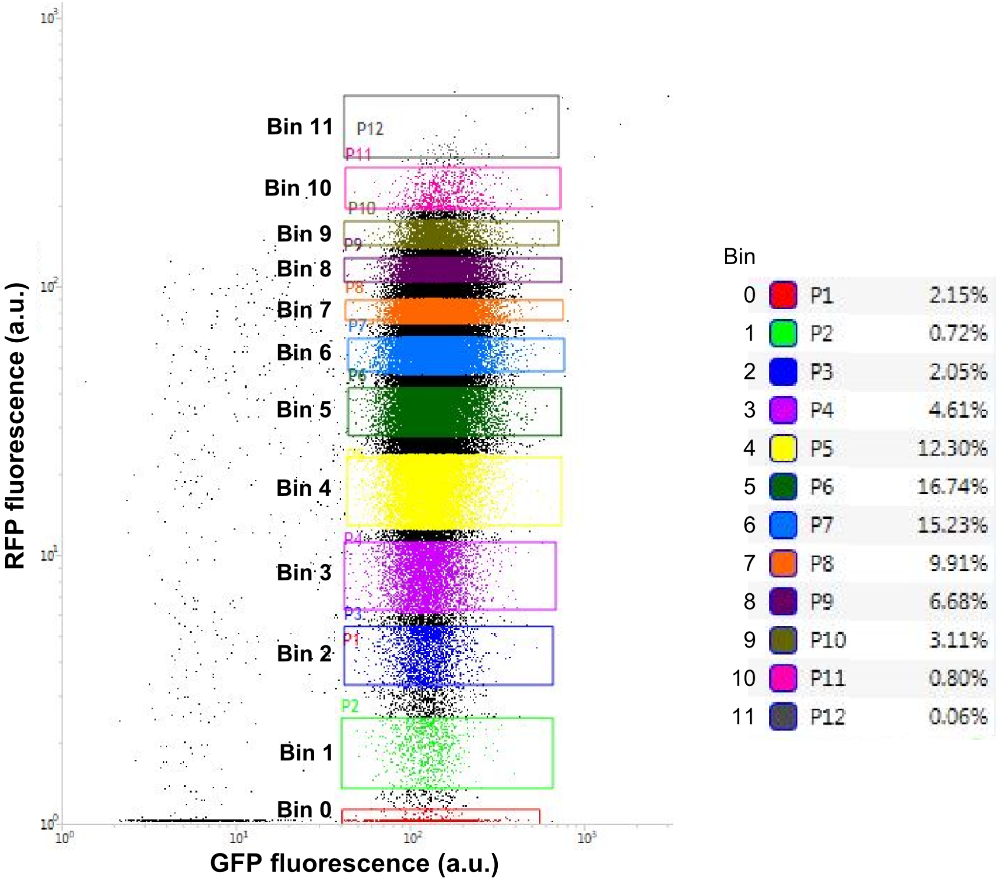

**Promoter library proD (σ^{70})**												
Bin	0	1	2	3	4	5	6	7	8	9	10	11
# sorted cells (x10^3)	47	13	40	80	230	300	300	230	120	60	15	2.6

Supplementary Figure 1: Fluorescence-activated cell sorting (FACS) scheme for the sigma factor 70 (σ^{70}) (of *Escherichia coli*)-specific promoter library of promoter proD [1]. The number of sorted cells for each bin are depicted below the scheme. (RFP: red fluorescent protein; GFP: green fluorescent protein; a.u.: arbitrary units).

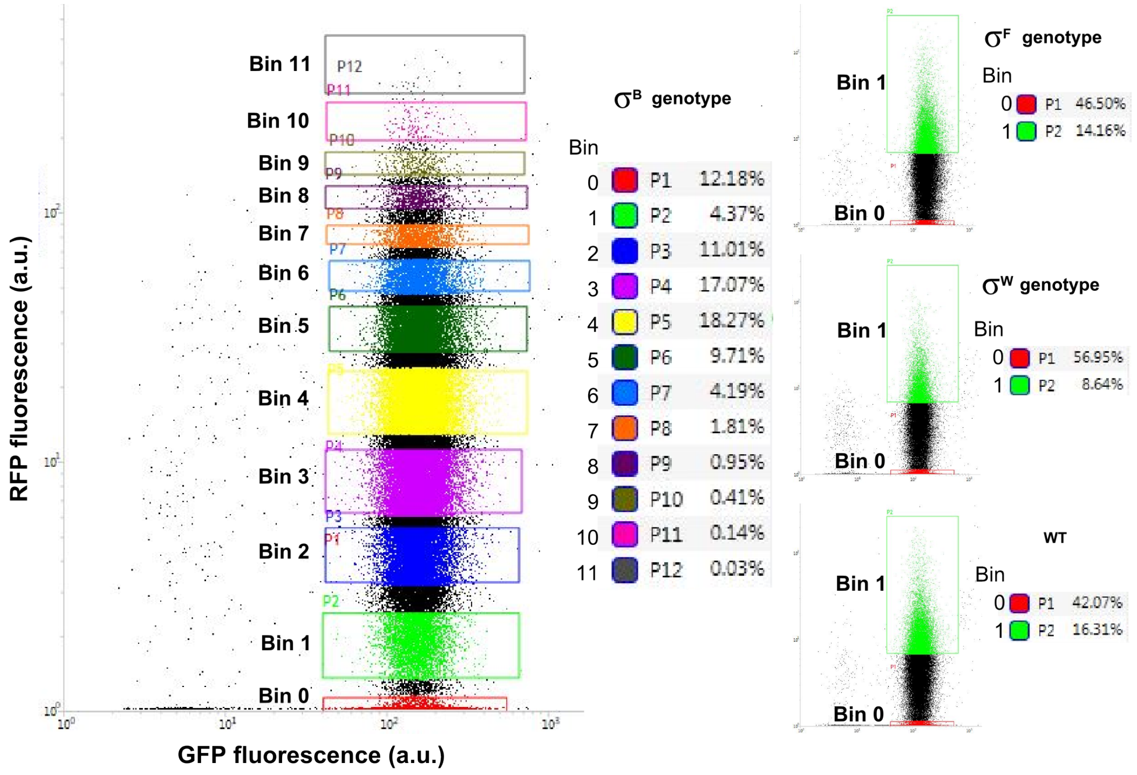

Promoter library B	σ^B genotype											
Bin	0	1	2	3	4	5	6	7	8	9	10	11
# sorted cells (x10^3)	130	50	120	18	180	100	45	25	14.2	6.9	7.7	1
	σ^F genotype			σ^W genotype			WT					
Bin	0	1		0	1		0	1				
# sorted cells (x10^3)	600	100		600	100		600	150				

Supplementary Figure 2: Fluorescence-activated cell sorting (FACS) scheme for the sigma factor B (σ^B) (of *Bacillus subtilis*)-specific promoter library B in *Escherichia coli*, in the presence of cognate σ^B or non-cognate σ^F or σ^W (*B. subtilis*) or only wild-type *E. coli* (WT) σs. The number of sorted cells for each bin is depicted below the scheme. (RFP: red fluorescent protein; GFP: green fluorescent protein; a.u.: arbitrary units).

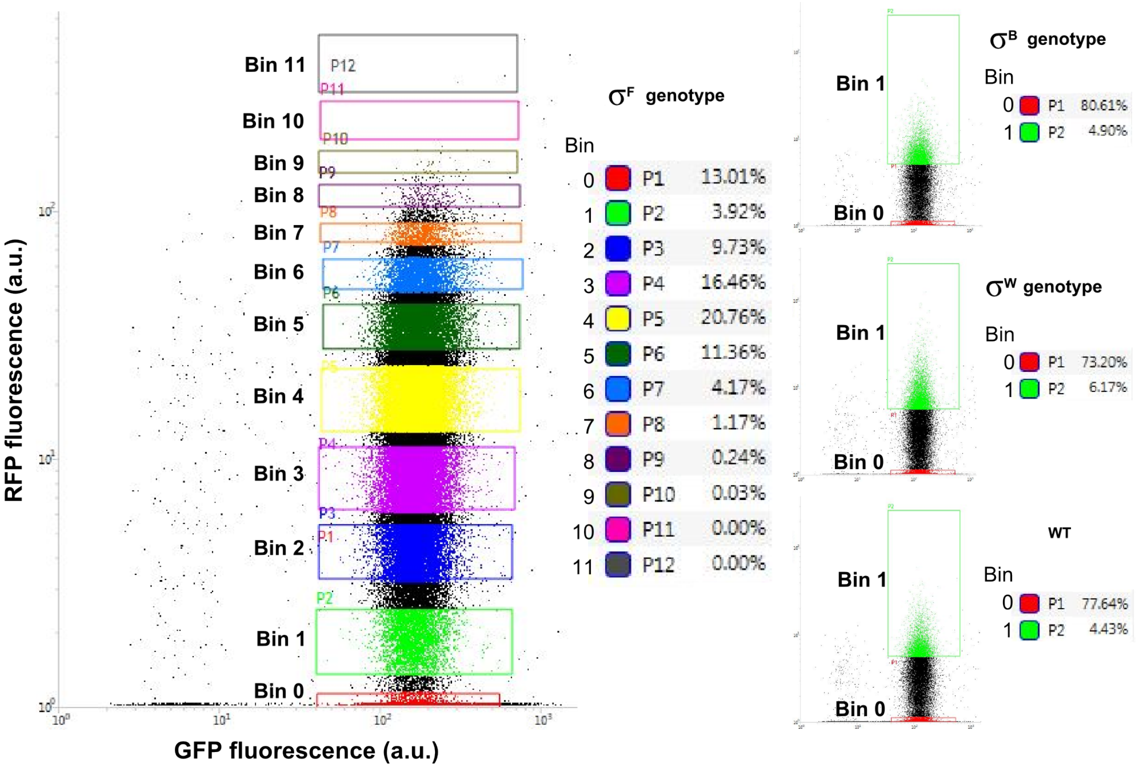

Promoter library F	σ^F genotype											
Bin	0	1	2	3	4	5	6	7	8	9	10	11
# sorted cells (x10^3)	250	80	190	320	400	250	80	25	8.2	1.1	0.5	0.061
	σ^B genotype			σ^W genotype			WT					
Bin	0	1		0	1		0	1				
# sorted cells (x10^3)	1600	100		1500	120		1500	100				

Supplementary Figure 3: Fluorescence-activated cell sorting (FACS) scheme for the sigma factor F (σ^F) (of *Bacillus subtilis*)-specific promoter library F in *Escherichia coli*, in the presence of cognate σ^F or non-cognate σ^B or σ^W (*B. subtilis*) or only wild-type *E. coli* (WT) σs. The number of sorted cells for each bin is depicted below the scheme. (RFP: red fluorescent protein; GFP: green fluorescent protein; a.u.: arbitrary units).

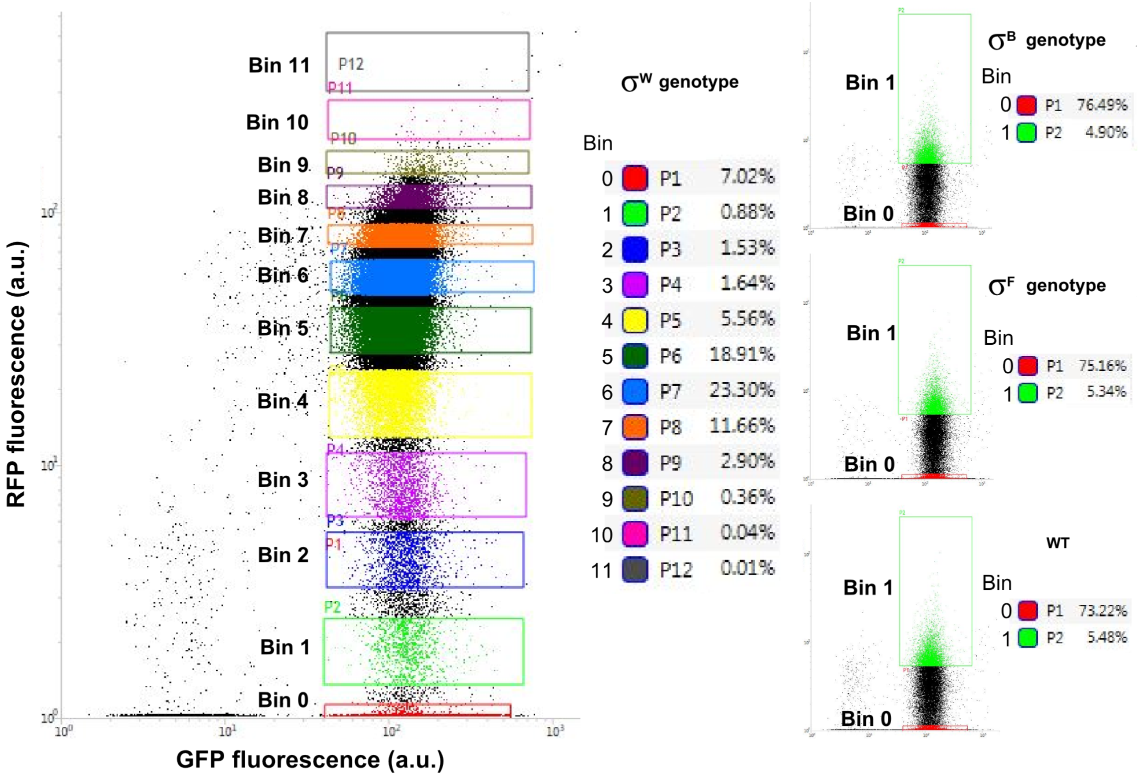

Promoter library W	σ^W genotype											
Bin	0	1	2	3	4	5	6	7	8	9	10	11
# sorted cells (x10^3)	110	15	25	25	90	300	370	170	250	130	50	5
	σ^B genotype			σ^F genotype			WT					
Bin	0	1		0	1		0	1				
# sorted cells (x10^3)	1200	80	1200	80			1200	80				

Supplementary Figure 4: Fluorescence-activated cell sorting (FACS) scheme for the sigma factor W (σ^W) (of *Bacillus subtilis*)-specific promoter library W in *Escherichia coli*, in the presence of cognate σ^F or non-cognate σ^B or σ^F (*B. subtilis*) or only wild-type *E. coli* (WT) σs. The number of sorted cells for each bin is depicted below the scheme. (RFP: red fluorescent protein; GFP: green fluorescent protein; a.u.: arbitrary units).

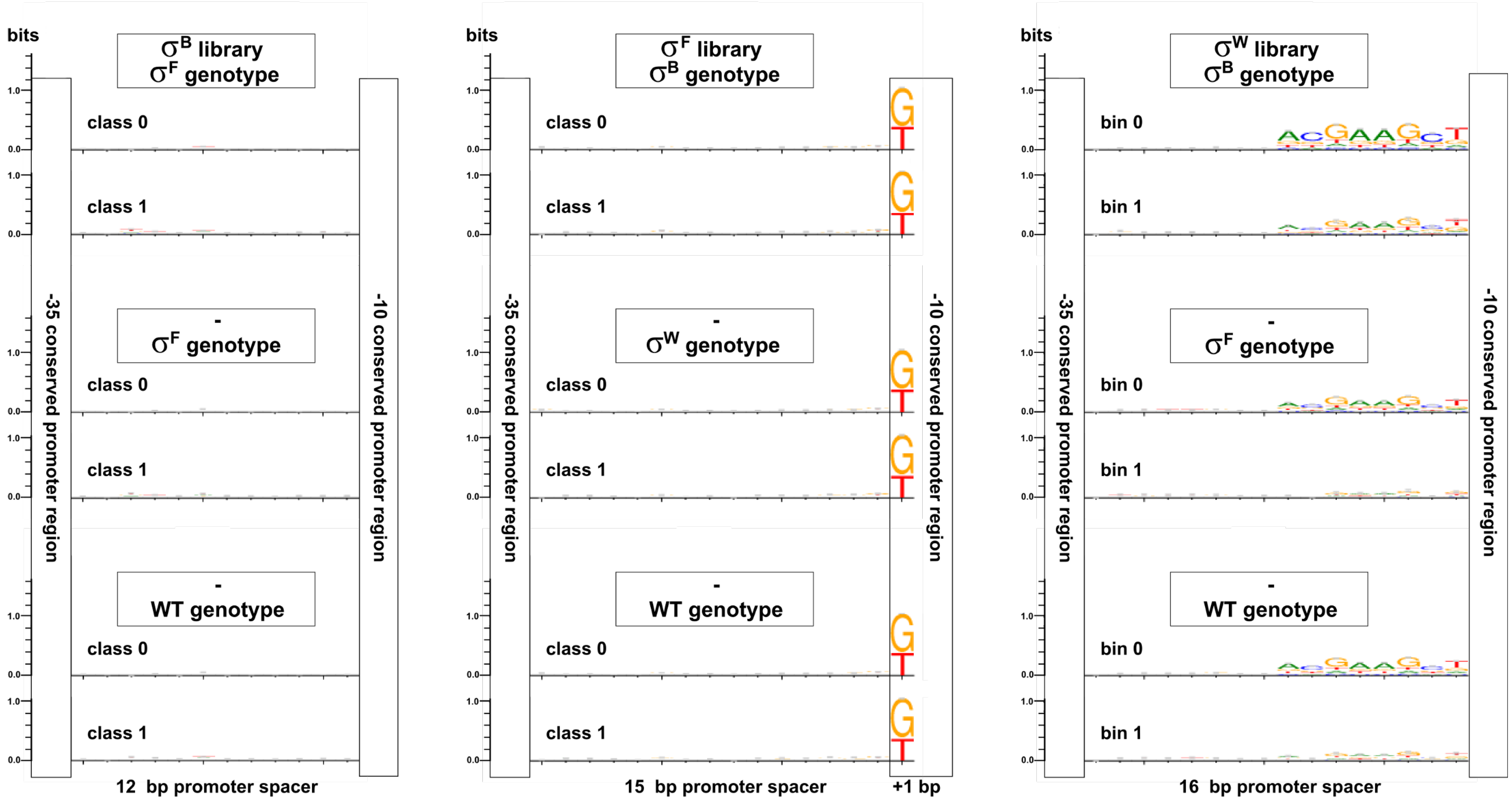

Supplementary Figure 5: Sequence logos illustrating the promoter spacer motifs, situated between the conserved -35 and -10 regions. These are given for each of the two classes to which the sequences of the sorted *Escherichia coli* sigma factor 70 (σ^{70}) and *Bacillus subtilis* σ^B, σ^F and σ^W-specific promoter libraries in presence of their non-cognate σs were assigned. In case of our σ^F library, one engineered base pair following the spacer is situated in the -10 conserved promoter region. Motifs were created with WebLogo3.6.0 [2], using the filtered data sets.

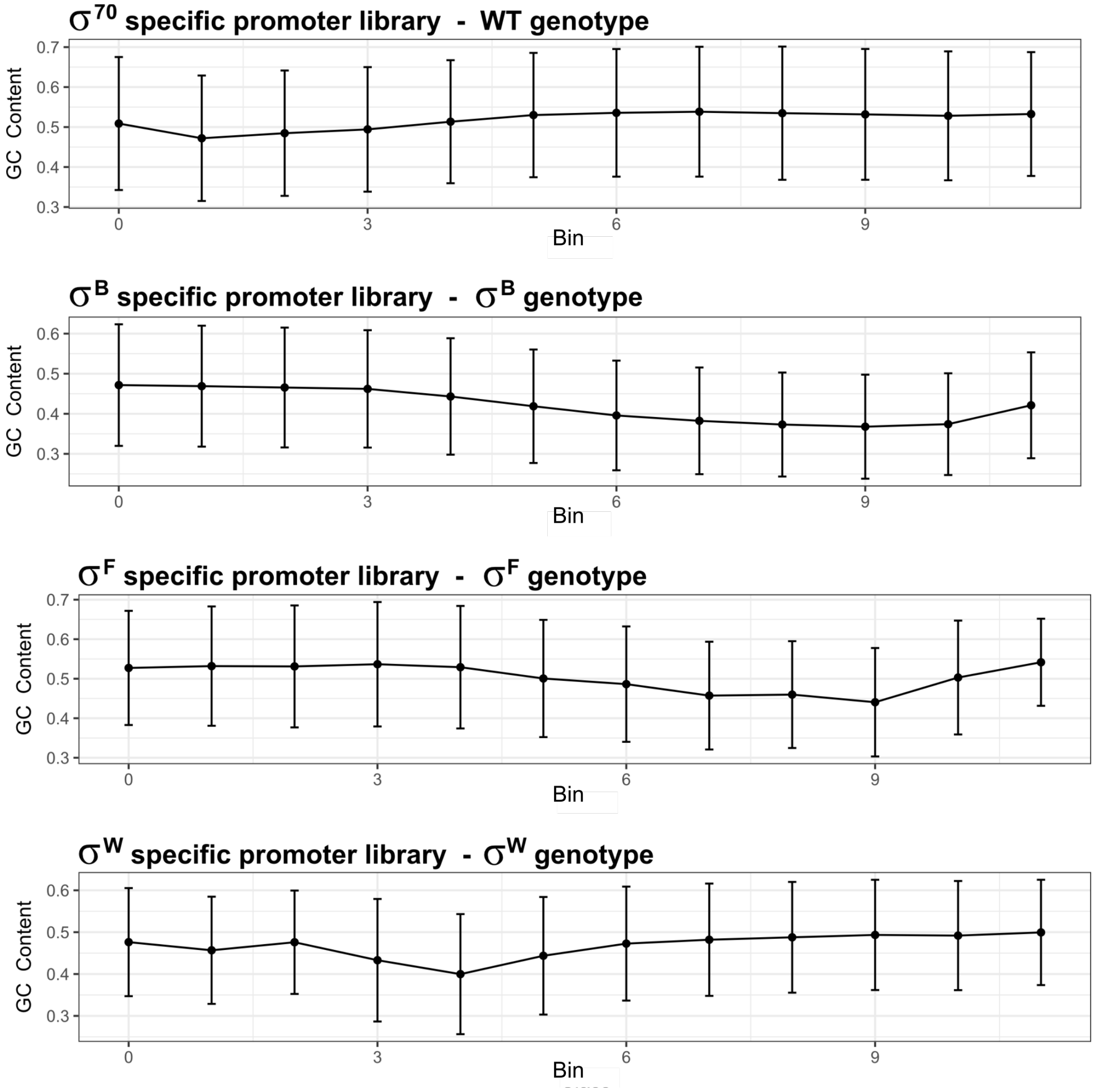

Supplementary Figure 6: GC content of the promoter spacer libraries for each set of sequences assigned to a specific bin. Data is given for the *Escherichia coli* sigma factor 70 (σ^{70}; n=285,762) and *Bacillus subtilis* σ^{B} (n=168,320) σ^{F} (n=251,146) and σ^{W} (n=187,091) specific promoter libraries, sorted in presence of their cognate σ. Data are represented as mean values +/- the standard deviation. (WT: wild-type). The spearman rank coefficient over all the bins for each promoter is 0.05 for σ^{70}, -0.18 for σ^{B}, -0.09 for σ^{F} and 0.04 for σ^{W} ($p<10^{-100}$). However, the Mann–Whitney U rejects the hypothesis that consecutive bins are sampled from the same distributions (data not shown, exceptions are bin 3-4 for σ^{70}, bin 0-1 for σ^{B}, bin 2-3 for σ^{F} and bin 9-10 for σ^{W}). As the means are not strictly ordered, GC-content of the promoters do not follow a linear relationship. Source data are provided as a Source Data file.

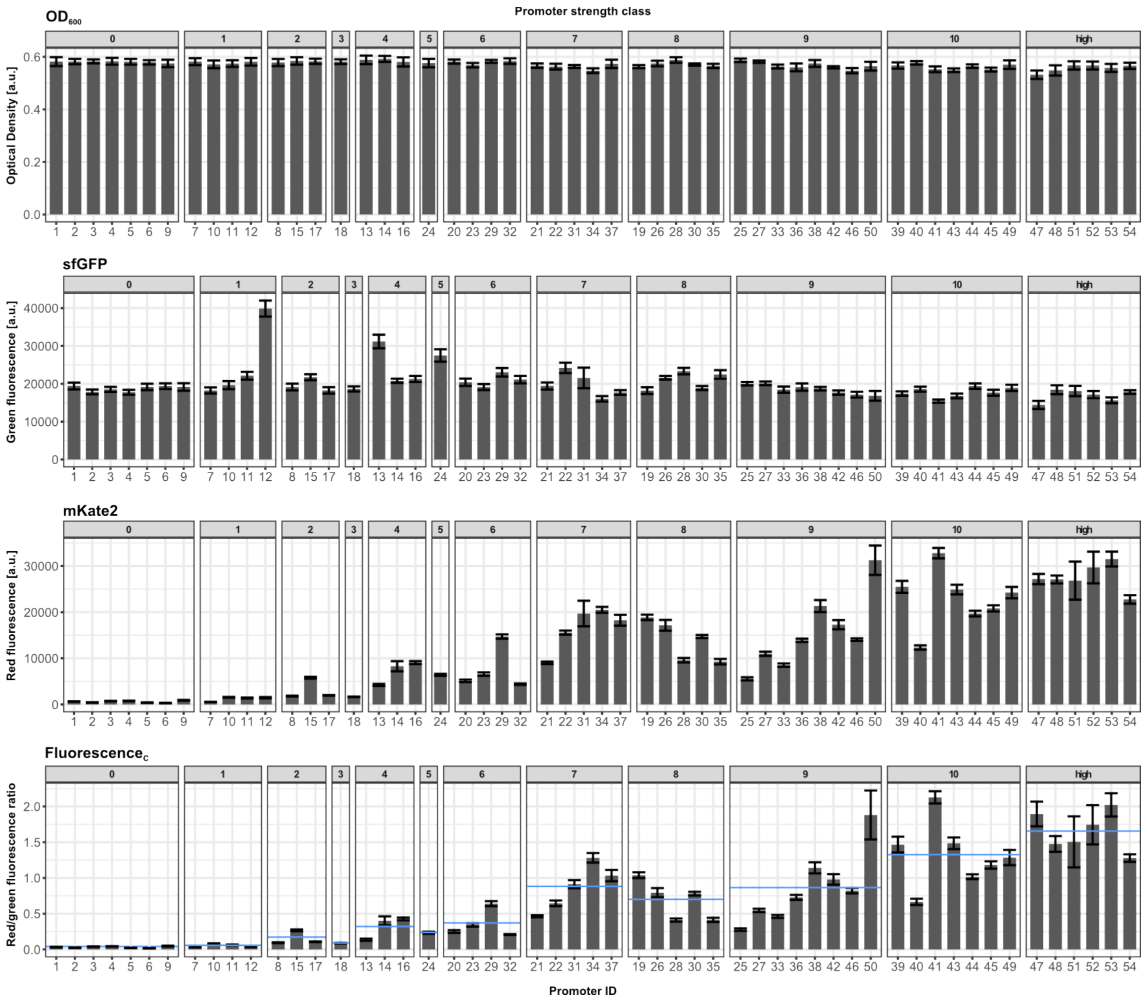

Supplementary Figure 7: Measured optical density (OD), sfGFP fluorescence, mKate fluorescence and the calculated corrected fluorescence (Materials and Methods) for the 54 forward engineered σ^{70}-specific promoters**.** Data are represented as mean values +/- the standard deviation derived from eight biological replicates. Blue horizontal lines in the bottom plot depict the mean promoter transcription initiation frequency (TIF) of the promoters within the predicted class. Source data are provided as a Source Data file.

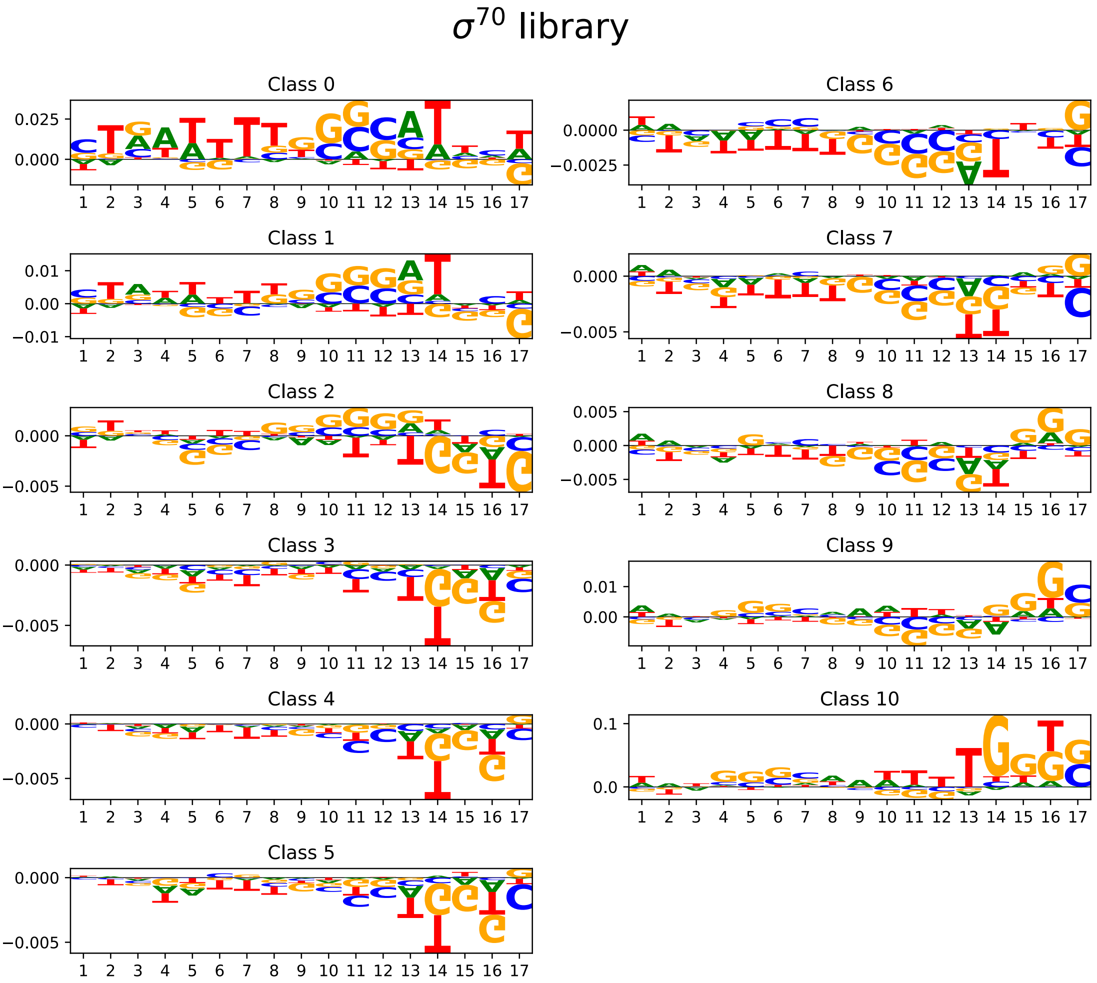

Supplementary Figure 8: Attribution scores obtained by DeepLIFT [3] for the model trained on σ^{70}-specific promoters. Data are represented as mean values for each sequence in the test set (n= 56,885 samples) and separated by class (following the class distributions listed in Supplementary Table 1) and position (x-axis).

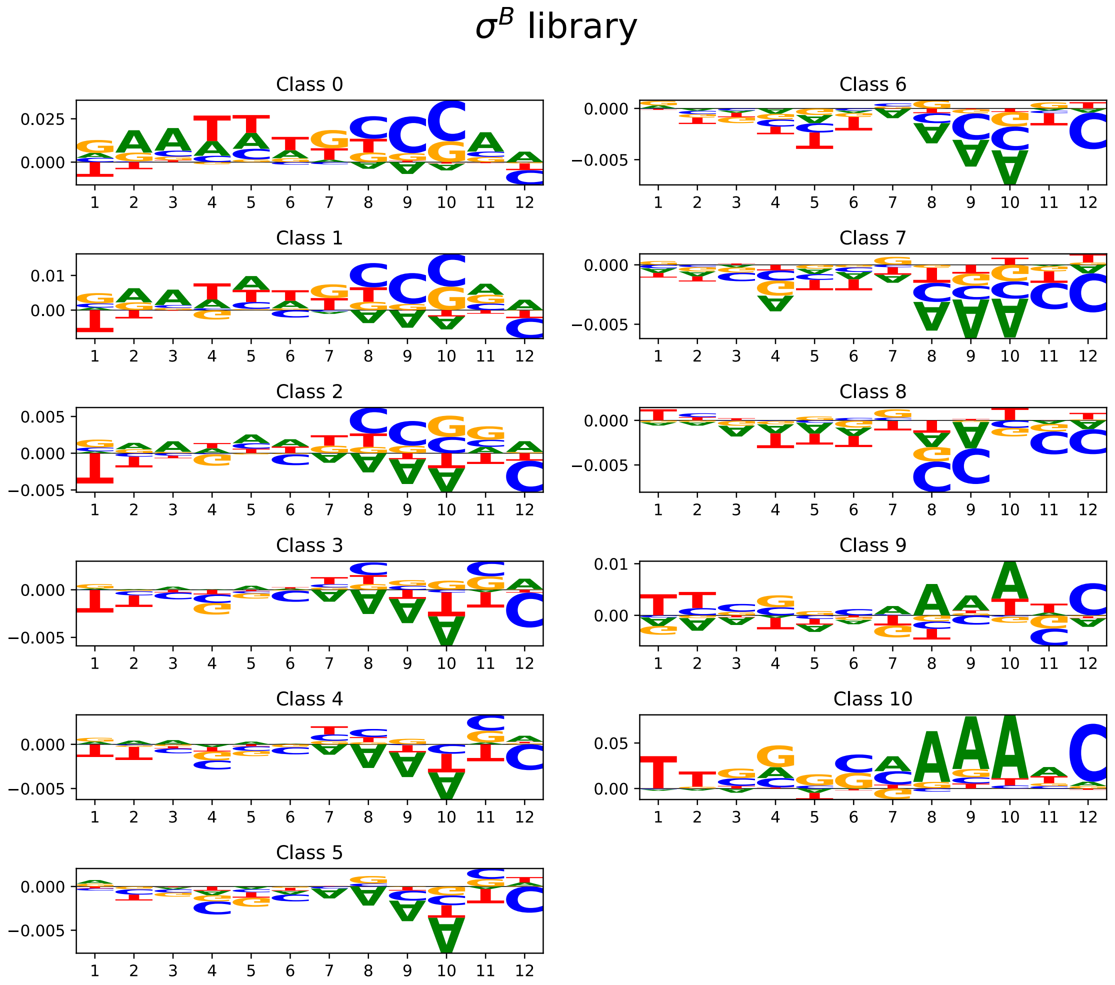

Supplementary Figure 9: Attribution scores obtained by DeepLIFT [3] for the model trained on σ^B-specific promoters. Data are represented as mean values for each sequence in the test set (n= 29,850 samples) and separated by class (following the class distributions listed in Supplementary Table 1) and position (x-axis).

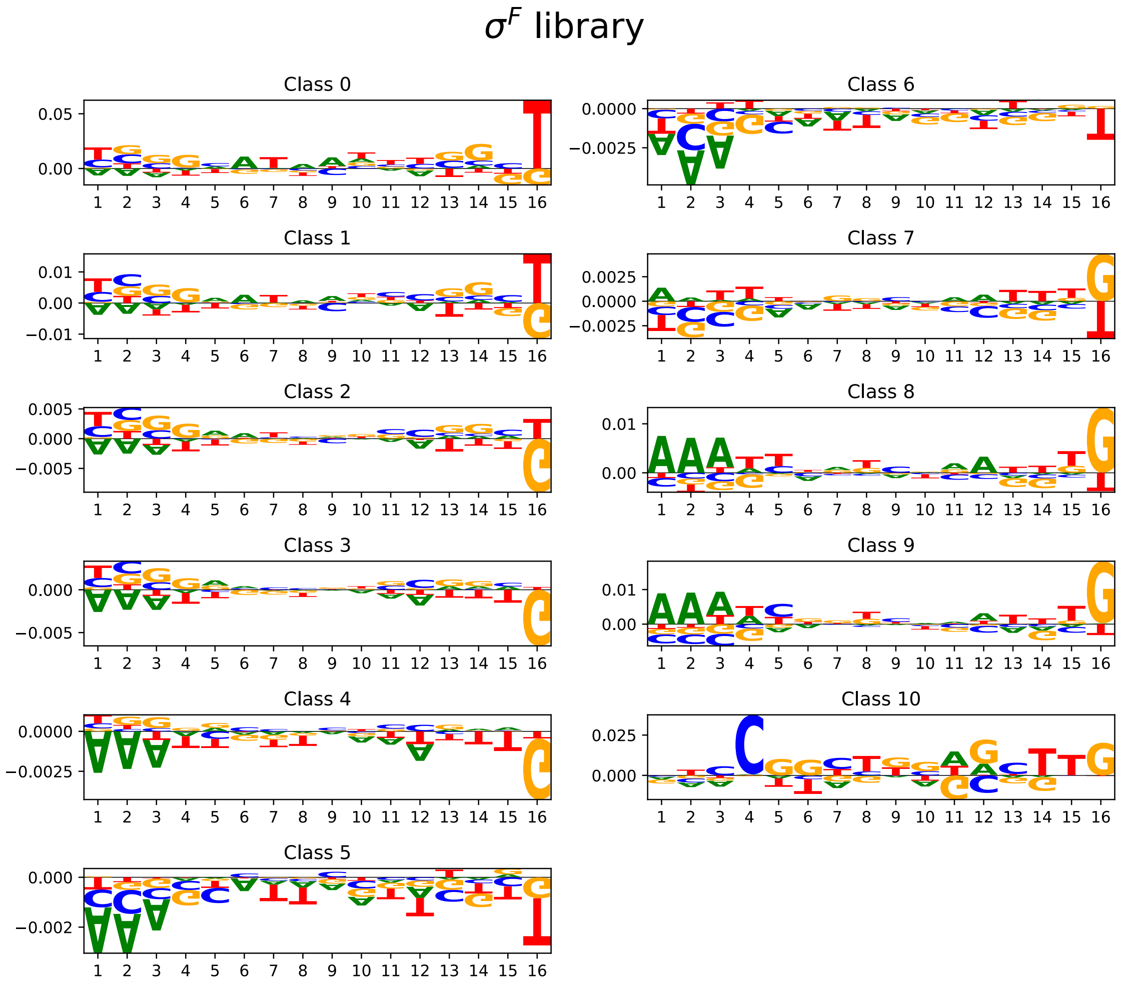

Supplementary Figure 10: Attribution scores obtained by DeepLIFT [3] for the model trained on σ^F-specific promoters. Data are represented as mean values for each sequence in the test set (n= 44,864 samples) and separated by class (following the class distributions listed in Supplementary Table 1) and position (x-axis).

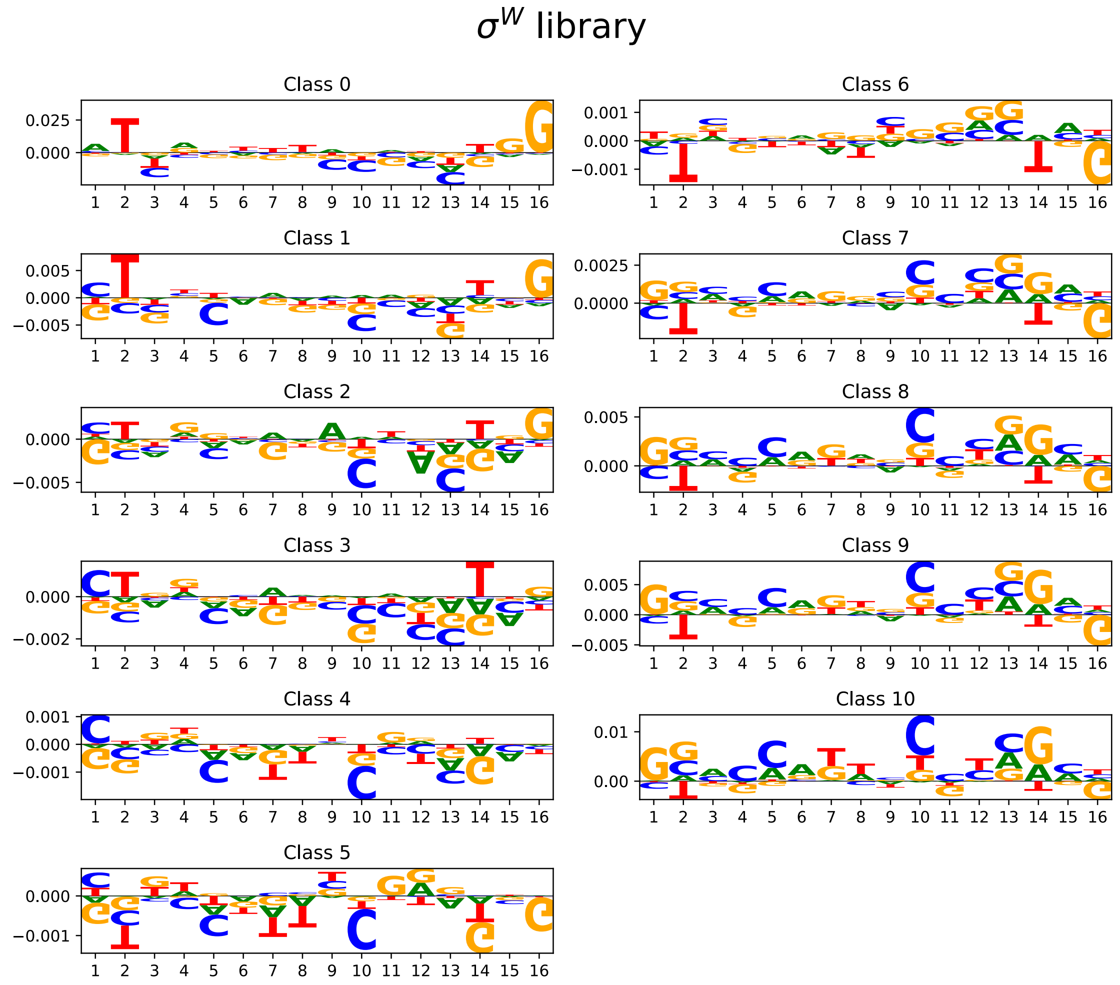

Supplementary Figure 11: Attribution scores obtained by DeepLIFT [3] for the model trained on σ^W-specific promoters. Data are represented as mean values for each sequence in the test set (n= 36,963 samples) and separated by class (following the class distributions listed in Supplementary Table 1) and position (x-axis).

References

1. Davis, J. H., Rubin, A. J. & Sauer, R. T. Design, construction and characterization of a set of insulated bacterial promoters. *Nucleic Acids Res.* **39,** 1131–1141 (2011).

2. Crooks, G. E., Hon, G., Chandonia, J.-M. & Brenner, S. E. WebLogo: A Sequence Logo Generator. *Genome Res.* **14,** 1188–1190 (2004).

3. Shrikumar, A., Greenside, P. & Kundaje, A. Learning Important Features Through Propagating Activation Differences. *arXiv:1704.02685 [cs.CV]* (2017).
