# Supplementary material for: Predictive design of sigma factor-specific promoters
Source: Nat Commun. 2020 Nov 16;11:5822. doi: 10.1038/s41467-020-19446-w (PMC7670410; doi:10.1038/s41467-020-19446-w)
Supplement: Supplementary file 3 — Reporting Summary [file 41467_2020_19446_MOESM3_ESM.pdf]

nature research

Corresponding author(s): Marjan De Mey

Last updated by author(s): Sep 20, 2020

Reporting Summary

Nature Research wishes to improve the reproducibility of the work that we publish. This form provides structure for consistency and transparency in reporting. For further information on Nature Research policies, see our Editorial Policies and the Editorial Policy Checklist.

Statistics

For all statistical analyses, confirm that the following items are present in the figure legend, table legend, main text, or Methods section.

n/a	Confirmed	
☐	☒	The exact sample size (*n*) for each experimental group/condition, given as a discrete number and unit of measurement
☐	☒	A statement on whether measurements were taken from distinct samples or whether the same sample was measured repeatedly
☐	☒	The statistical test(s) used AND whether they are one- or two-sided<br>*Only common tests should be described solely by name; describe more complex techniques in the Methods section.*
☒	☐	A description of all covariates tested
☒	☐	A description of any assumptions or corrections, such as tests of normality and adjustment for multiple comparisons
☐	☒	A full description of the statistical parameters including central tendency (e.g. means) or other basic estimates (e.g. regression coefficient) AND variation (e.g. standard deviation) or associated estimates of uncertainty (e.g. confidence intervals)
☒	☐	For null hypothesis testing, the test statistic (e.g. *F*, *t*, *r*) with confidence intervals, effect sizes, degrees of freedom and *P* value noted<br>*Give P values as exact values whenever suitable.*
☒	☐	For Bayesian analysis, information on the choice of priors and Markov chain Monte Carlo settings
☒	☐	For hierarchical and complex designs, identification of the appropriate level for tests and full reporting of outcomes
☐	☒	Estimates of effect sizes (e.g. Cohen's *d*, Pearson's *r*), indicating how they were calculated

Our web collection on statistics for biologists contains articles on many of the points above.

Software and code

Policy information about availability of computer code

Data collection	Duplexed DNA design: ViennaRNA (2.4.11), RNAcofold package (2.4.11)<br>FACS data: BD FACS Sortware sorter software<br>Fluorescence and OD data on MTP-scale: Tecan i-control (1.10) and Magellan software (Magellan tracker 7.2)
Data analysis	Sequence analysis: WebLogo-3.6.0<br>Machine learning: Python-3.7.8 (numpy-1.19.1, pandas-1.1.0, PyTorch 1.2.0)<br>Custom promoter strength tool: https://github.com/MEMO-group/ProD (1.0)<br>In vivo validation test of the machine learning tool: R script (R 3.2.3)

For manuscripts utilizing custom algorithms or software that are central to the research but not yet described in published literature, software must be made available to editors and reviewers. We strongly encourage code deposition in a community repository (e.g. GitHub). See the Nature Research guidelines for submitting code & software for further information.

Data

Policy information about availability of data

All manuscripts must include a data availability statement. This statement should provide the following information, where applicable:

- Accession codes, unique identifiers, or web links for publicly available datasets
- A list of figures that have associated raw data
- A description of any restrictions on data availability

EMBL-EBI ArrayExpress E-MTAB-8734

Field-specific reporting

Please select the one below that is the best fit for your research. If you are not sure, read the appropriate sections before making your selection.

- [x] Life sciences
- [ ] Behavioural & social sciences
- [ ] Ecological, evolutionary & environmental sciences

For a reference copy of the document with all sections, see nature.com/documents/nr-reporting-summary-flat.pdf

Life sciences study design

All studies must disclose on these points even when the disclosure is negative.

Sample size	FACS data (machine learning): No sample-size calculation performed. The use of fluorescent activated cell sorting was argumented as it results in large sample sizes.<br>In vivo validation set (spearman rank correlation test): In order to reject the Null hypothesis using a spearman rank test, the required sample size was determined based on the error rate of the machine learning tool on the test set.
Data exclusions	FACS data (machine learning):Exclusion of the samples recovered from the flow cytometry bin covering the cells with zero to low fluorescence. This is done as cells with no expression of the fluorescence protein might have defects. This was established after evaluation of the data.<br>In vivo validation set (spearman rank correlation test): No data were excluded.
Replication	FACS data (machine learning): a large variety of populations with vectors containing randomized spacer sequences was created (12) and grown. These were all applied for binning using FACS and sequencing using MiSeq. No replications were performed due to the large investment required to obtain these.<br>In vivo validation set (spearman rank correlation test): 8 replicates were successfully created and applied for data processing.
Randomization	Machine learning: Sequences are randomized before binning by the flow cytometer.<br>In vivo validation test of the machine learning tool: Monte Carlo was used to generate random sequences that were classified based on predicted strength.
Blinding	The study does not perform subjective trials or evaluations. Blinding has therefore not been performed.

Reporting for specific materials, systems and methods

We require information from authors about some types of materials, experimental systems and methods used in many studies. Here, indicate whether each material, system or method listed is relevant to your study. If you are not sure if a list item applies to your research, read the appropriate section before selecting a response.

Materials & experimental systems

n/a	Involved in the study	
[x]	[ ]	Antibodies
[x]	[ ]	Eukaryotic cell lines
[x]	[ ]	Palaeontology and archaeology
[x]	[ ]	Animals and other organisms
[x]	[ ]	Human research participants
[x]	[ ]	Clinical data
[x]	[ ]	Dual use research of concern

Methods

n/a	Involved in the study	
[x]	[ ]	ChIP-seq
[x]	[ ]	Flow cytometry
[x]	[ ]	MRI-based neuroimaging
